# Automated Framework for the Inclusion of a His–Purkinje System in Cardiac Digital Twins of Ventricular Electrophysiology

**DOI:** 10.1007/s10439-021-02825-9

**Published:** 2021-08-24

**Authors:** Karli Gillette, Matthias A. F. Gsell, Julien Bouyssier, Anton J. Prassl, Aurel Neic, Edward J. Vigmond, Gernot Plank

**Affiliations:** 1 Gottfried Schatz Research Center Biophysics, Medical University of Graz, Graz, Austria; 2 BioTechMed-Graz, Graz, Austria; 3 IHU Liryc, Electrophysiology and Heart Modeling Institute, Fondation Bordeaux Université, Pessac-Bordeaux, France; 4 NumeriCor GmbH, Graz, Austria

**Keywords:** His–Purkinje system, Forward ECG modeling, Parameter identification, Computational cardiac modeling, Electrocardiogram

## Abstract

Personalized models of cardiac electrophysiology (EP) that match clinical observation with high fidelity, referred to as cardiac digital twins (CDTs), show promise as a tool for tailoring cardiac precision therapies. Building CDTs of cardiac EP relies on the ability of models to replicate the ventricular activation sequence under a broad range of conditions. Of pivotal importance is the His–Purkinje system (HPS) within the ventricles. Workflows for the generation and incorporation of HPS models are needed for use in cardiac digital twinning pipelines that aim to minimize the misfit between model predictions and clinical data such as the 12 lead electrocardiogram (ECG). We thus develop an automated two stage approach for HPS personalization. A fascicular-based model is first introduced that modulates the endocardial Purkinje network. Only emergent features of sites of earliest activation within the ventricular myocardium and a fast-conducting sub-endocardial layer are accounted for. It is then replaced by a topologically realistic Purkinje-based representation of the HPS. Feasibility of the approach is demonstrated. Equivalence between both HPS model representations is investigated by comparing activation patterns and 12 lead ECGs under both sinus rhythm and right-ventricular apical pacing. Predominant ECG morphology is preserved by both HPS models under sinus conditions, but elucidates differences during pacing.

**Supplementary Information:**

The online version contains supplementary material available at 10.1007/s10439-021-02825-9.

## Introduction

Anatomically and functionally personalized models of cardiac electrophysiology (EP) are considered a promising complementary tool for tailoring future cardiac precision therapies^7^,^10^ and for the cost-effective, safe, and ethical testing of device therapies.^33^ Such digital replicas of a patient’s heart that match all available clinical observations with high fidelity within the bounds of clinical data uncertainty are referred to as cardiac digital twin (CDTs). CDTs of cardiac EP built on first principles offer predictive potential, owing to their mechanistic nature and their ability to quantitatively integrate clinical data to achieve specificity for a given individual. Building CDTs from clinical data remains to be a challenging problem. Recent methodological advances in terms of both anatomical and functional twinning workflows suggest that streamlined generation of CDTs at scale is becoming feasible.^4^,^13^,^21^,^28^

Of pivotal importance for a sound mechanistic representation of cardiac EP is the consideration of effects mediated by the His–Purkinje system (HPS) in the ventricles. Computational models that integrate *a priori* knowledge of the HPS and replicate QRS complexes in all electrocardiogram (ECG) leads can be considered likely and plausible approximations of a given patient’s EP. Integrating models of the HPS in models of human ventricular EP and tailoring these to create a CDT is complicated by two major factors. First, there is limited knowledge about the actual topology of the HPS, its variability within the general human population,^9^ and the micro-anatomical structure of the purkinje ventricular junctions (PVJs) that couple the HPS with the myocardium.^32^ Secondly, the generation of a topologically-detailed HPS in ventricular models of EP is not trivial and often computationally expensive due to the structural, spatially-varying complexity of such networks. Alteration of HPS parameters controlling density, topological features, and areas of PVJs to minimize the discrepancy between modeled and measured ECGs for personalization therefore appears a daunting endeavor. While automated methods for generating models of HPS do exist with varying complexity,^21^,^25^,^31^ such methods may fail to produce a physiological QRS complex or are not yet suitable for patient-specific parameterization within digital twinning pipelines.

Personalized models of the HPS are thus needed for building CDTs that are capable of capturing ventricular EP mechanisms under a broad range of conditions. To achieve clinical feasibility, workflows for building models of the HPS must achieve a high degree of automation to facilitate the robust production of models at scale. To be credible, CDTs must be able to replicate QRS complexes observed in body surface potential maps or in the clinically standard 12 lead ECG with sufficiently high morphological fidelity.

We introduce an automated two stage approach for generating HPSs of high-fidelity for use in digital twinning pipelines. First, an abstract general representation of emergent features of the HPS was formulated and personalized in a previous study^13^ based on the notion of earliest activation sites (EASs) mediated by the fascicles of the HPS and the sub-endocardial (SE) domain where they reside. At a second stage, as described in detail in this study, the phenomenological lightweight fascicle-based model is replaced by a computationally more costly HPS model constructed using a physiologically-relevant Purkinje model. The Purkinje model builds upon and improves the previously reported method in Ref. 25. The feasibility of automatically replacing a simplified fascicle-based HPS model by a topologically realistic HPS model with a complex Purkinje network is demonstrated in a single subject under both sinus and pacing conditions.

A model for the subject was created from clinical magnetic resonance images (MRIs) and fitted with an anatomical reference frame allowing automated control of all model parameters relating to both the architecture of the HPS, as well as ventricular EP. Discrepancies within the ventricular activation and concomitant 12 lead ECG between the topologically realistic Purkinje model featuring antidromic activation and the simpler fascicle-based model, accounting for HPS effects in a phenomenological sense, are elucidated by simulating both healthy sinus rhythm and right ventricular (RV) apical pacing. Sinus rhythm is also analyzed in terms of the goodness of fit with the measured 12 lead ECG.

## Materials and Methods

### Model Generation from Clinical MR Images

As part of an MRI study approved by the ethics review board of the Medical University of Graz (EKNr: 24-126 ex 11/12), MRI data consisting of both a torso and iso-volumetric 3D whole heart were acquired for a healthy volunteer. The volunteer was male of 47 years of age. A 12 lead ECG was recorded using MRI-compatible electrodes at the time of the study at ${2000}\hbox { Hz}$ using a clinical-based system. The 12 lead ECG was subsequently filtered using a ${50}\hbox { Hz}$ notch filter, ${0.5}\hbox { Hz}$ high-pass filter, and a low pass filter of 60 Hz in accordance with the clinical standards. Segmentation of the torso MRI was performed within publicly available software *Seg3D*.^6^ The 3D whole heart cardiac MRI was segmented automatically using a convolutional neural network^22^ followed by manual corrections in *Seg3D* when required. Registration of the heart into the torso was also performed within *Seg3D* using a modified iterative closest point algorithm. An anatomically-accurate multi-label finite element model was then generated at an average spatial resolution of the cardiac domain of $\,\sim$1 mm. Electrode placements were recovered from the torso MRI and localized to the nearest node within the mesh.

### Anatomical Reference Frame

To automatically control spatially-varying entities of ventricular EP, such as locations of the EASs, pacing sites, or the SE domain, an anatomical reference frame ${\mathcal {X}}$ was introduced to provide anatomically meaningful descriptions of location and direction within the ventricles. As thoroughly detailed in Ref. 13, the reference frame utilizes universal ventricular coordinates (UVCs)^2^ to define a bijective function ${\mathcal {B}}_{\mathrm{UVC}} : \Omega _{\mathrm{biv}}\rightarrow D_{_{\mathrm {UVC}}}$, mapping between the Cartesian model $\Omega _{\mathrm{biv}}$ and the abstract UVC space $D_{_{\mathrm {UVC}}}$. Each coordinate $\mathbf {b} = (z, \rho , \varphi , \nu ) \in D_{_{\mathrm {UVC}}}$ comprises an apicobasal (*z*), transmural ($\rho$), rotational ($\varphi$) and ventricular ($\nu$) component and there exists a unique $\mathbf {x} \in \Omega _{\mathrm{biv}}$, such that $\mathbf {b} = {\mathcal {B}}_{\mathrm{UVC}}(\mathbf {x})$ as well as $\mathbf {x} = {\mathcal {B}}_{\mathrm{UVC}}^{-1}(\mathbf {b})$ holds. A *k*d-tree based tool was implemented within the open source mesh manipulation software *meshtool*^20^ to realize the mapping ${\mathcal {B}}_{\mathrm{UVC}}$.

### Configuration of the His–Purkinje System

**Figure 1:**
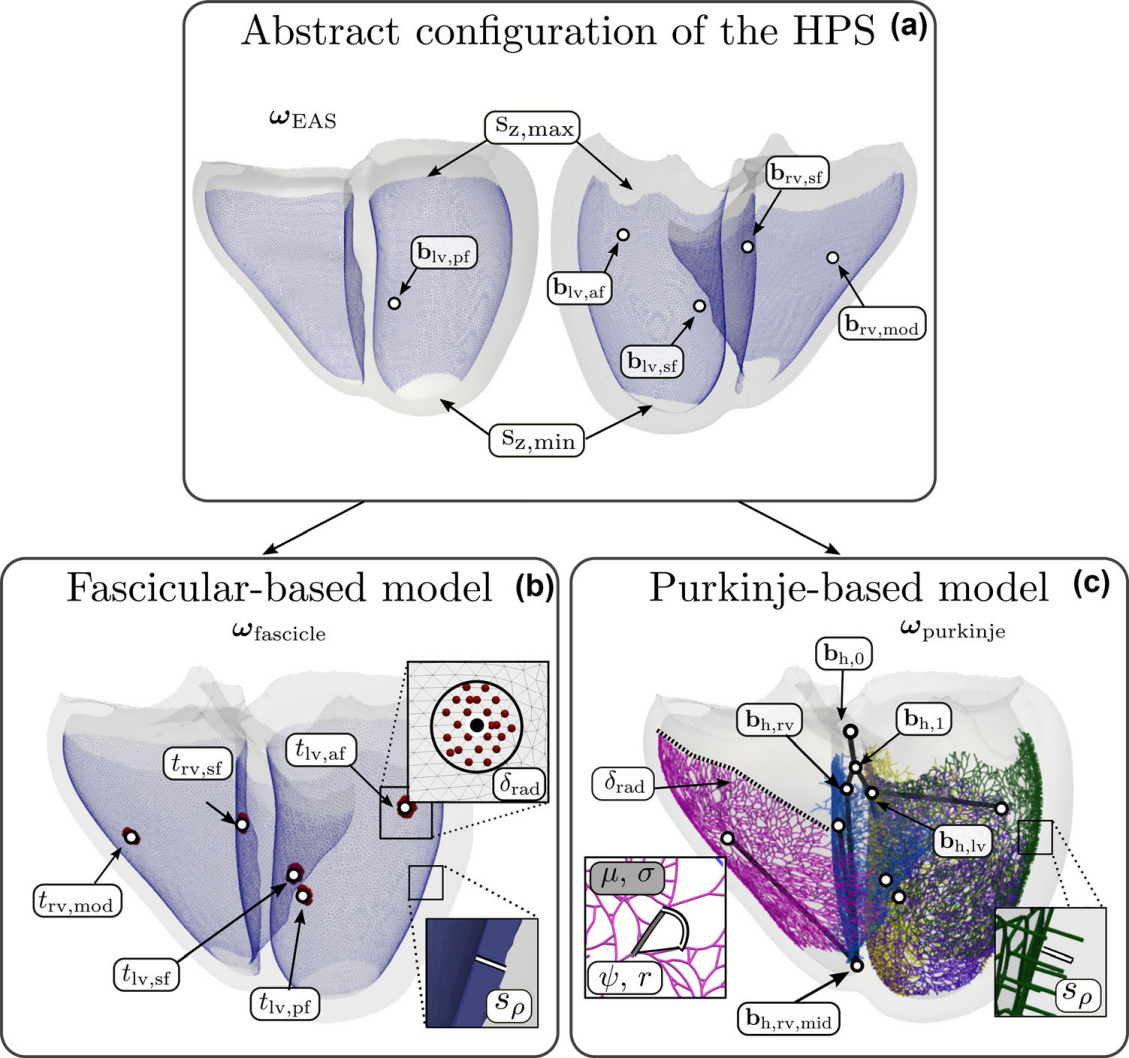
Parameter definition of the HPS. (a) Top panel shows the representation of the EASs and SE layer bounded apicobasally within $\varvec{\omega }_{\mathrm {EAS}}$. (b) The left bottom panel shows the fascicular-based model that defines discs with a given radius $\delta _{{\mathrm{rad}}}$ centered around root locations corresponding to EASs fired at given timings defined by $\mathbf {t}_{\mathrm {his}}$. (c) The Purkinje-based model requires definition of bifurcation points $\mathbf {b}$ of the His-bundle and fasicular network. Purkinje trees are grown from root locations corresponding to the EASs with given growth and branching parameters to a given sizing dictated by $\delta _{{\mathrm{rad}}}$.

Both fascicular- and Purkinje network-based models build upon the same definition of fascicular root locations that determine the ventricular EASs (see Fig. 1). Based on experimental studies^11^ the HPS was assumed to have $N=5$ EASs, three pertaining to the septal, anterior and posterior fascicles in the left ventricle (LV) $(\mathbf {b}_{\mathrm {lv,sf}},\, \mathbf {b}_{\mathrm {lv,af}},\, \mathbf {b}_{\mathrm {lv,pf}})$, and two pertaining to septal and moderator band fascicles in the RV $(\mathbf {b}_{\mathrm {rv,sf}},\, \mathbf {b}_{\mathrm {rv,mod}})$. Each root location is defined by $\mathbf {b}_{\mathrm {lv,sf}}$, $\mathbf {b}_{\mathrm {lv,af}}$, $\mathbf {b}_{\mathrm {lv,pf}}$, $\mathbf {b}_{\mathrm {rv,sf}}$ and $\mathbf {b}_{\mathrm {rv,mod}}$ as an element in the UVC space $D_{_{\mathrm {UVC}}}$ within the reference frame ${\mathcal {X}}$.

The domain comprising Purkinje network and PVJs was constrained within both ventricles to a thin SE layer of bounded apicobasal extent. These bounds were defined by $\mathrm {s}_{\mathrm{z,min}} \in [0, 1]$ and $\mathrm {s}_{\mathrm{z,max}} \in [0, 1]$ with $\mathrm {s}_\mathrm{z,min} < \mathrm {s}_{\mathrm{z,max}}$. The transmural extent of the SE layer covered by the Purkinje network was limited to a transmural extent of $\mathrm {s}_{{\rho }} \in [0, 1]$. Outside this bounded SE layer, primarily in the apical region and close to the ventricular base, the HPS is assumed to be less abundant, or not present at all.^1^

Fascicular branches and the fast-conducting SE layer of the HPS are thus encapsulated by $\varvec{\omega }_{\mathrm {EAS}}$ with

$$\begin{aligned} \varvec{\omega }_{\mathrm {EAS}} = \left( \mathbf {b}_{\mathrm {lv,sf}},\, \mathbf {b}_{\mathrm {lv,af}},\, \mathbf {b}_{\mathrm {lv,pf}},\, \mathbf {b}_{\mathrm {rv,sf}},\, \mathbf {b}_{\mathrm {rv,mod}},\;\; \mathrm {s}_{\mathrm{z,min}},\, \mathrm {s}_{\mathrm{z,max}},\, \mathrm {s}_{{\rho }} \right) . \end{aligned}$$

While no patient-specific information of the HPS is inherently known, an iterative sampling was used to identify EASs defined within $\varvec{\omega }_{\mathrm {EAS}}$ that minimize the misfit between predicted and measured ECGs utilizing an ECG forward model able to compute ECGs at high biophysical fidelity with close to real-time performance as described in our previous work.^13^ A summary of the reported locations are available in Table 1. The SE layer in both models was confined between $\mathrm {s}_\mathrm{z,min}=0.15$ and $\mathrm {s}_{\mathrm{z,max}}=0.9$ apicobasally based on experimental measurements in Ref.^1^. A transmural extent of $\mathrm {s}_{\rho }=0.1$ was prescribed corresponding to approximately a single-element layer in the mesh.

**Table 1:** Baseline configuration for the root locations of the bundle branches that govern EASs and a SE layer bounded apicobasally within the HPS system.

Entity	Parameter	Value			
		UVC coordinates			
		*z*	$\rho$	$\varphi$	$\nu$
Earliest activation sites	$\mathbf {b}_{\mathrm {lv,sf}}$	0.61	0.0	0.73	− 1
	$\mathbf {b}_{\mathrm {lv,pf}}$	0.47	0.0	− 1.36	− 1
	$\mathbf {b}_{\mathrm {lv,af}}$	0.82	0.0	1.94	− 1
	$\mathbf {b}_{\mathrm {rv,sf}}$	0.73	1.0	− 0.04	− 1
	$\mathbf {b}_{\mathrm {rv,mod}}$	0.63	0.0	0.21	+ 1
SE layer	${s}_{\mathrm{z,min}}$	0.15			
	${s}_{\mathrm{z,max}}$	0.9			
	${s}_{{\rho }}$	0.1			

Root locations were optimized to generate comparable ECG morphology during sinus rhythm for the single subject as detailed in Ref. 13

### Fascicular-based Model

Root locations were embedded in the SE layer and assumed to be disc-like structures of a given radius $\delta _{\mathrm {rad}} > 0$ that couple the fascicles to the ventricular myocardium.

Conduction properties within the SE layer were assigned to facilitate the fast spread of activation modulated by the HPS. Timings for the fascicular terminals $\mathbf {t}_{\mathrm{his}} = (t_\mathrm{lv,af},\, t_{\mathrm{lv,sf}},\, t_{\mathrm{lv,pf}},\, t_{\mathrm{rv,sf}},\, t_\mathrm{rv,mod})$ could be prescribed to control activation timings of EASs within $\varvec{\omega }_{\mathrm {EAS}}$. All entities relating to the fascicular-based model of the HPS could then be described by a parameter vector $\varvec{\omega }_{\mathrm {{fascicle}}}$ given as

$$\begin{aligned} \varvec{\omega }_{\mathrm {fascicle}} = \left( \delta _{\mathrm {rad}},\;\; \mathbf {t}_{\mathrm {his}} \right) . \end{aligned}$$

### Purkinje-based Model

Purkinje networks were grown from every EAS using an algorithm that initially grew branches as seen in Ref. 25 but then used a fractal approach^14^ to produce a recombinant network. In brief, each Purkinje network was first initiated with a branch created from a starting node pertaining to each root location specified by $\varvec{\omega }_{\mathrm{EAS}}$. A maximum of two new branches are grown from every existing last node of previous generated branches and the operation is repeated for a given number of growth steps. Here, we mapped the number of growth steps and SE coverage to a parameter $\delta _{\mathrm {cov}} \in [0, 1]$. $\delta _{\mathrm {cov}}=1$ indicates complete coverage of the SE layer by the given bundle branch and $\delta _{\mathrm {cov}}=0$ indicates no growth. Each branch within the base Purkinje network is of a random length taken from a normal Gaussian distribution of mean $\mu$ and standard deviation $\sigma$. The direction of the branch is controlled by a given angle $\psi$ and a repulsion factor *r*.

Purkinje networks grown on the endocardial surface were assumed to have an intertwined mesh topology by allowing later merging of branches. Accordingly, given growth of a new branch into an existing portion of the Purkinje network, each branch was either completed or allowed to collide into an existing node, in which case the branch was connected to the node. No restriction on the number of collisions was defined, but every node in the network was allowed a maximum of two parents and two offspring branches. Note that each Purkinje network associated with a given fascicle is independent such that no merging between networks was allowed.

After the network growing terminated, PVJs were added. First, based on $\delta _{\mathrm cov}$, a linear PVJ density was computed for the network given the sum of all Purkinje cable lengths. The network nodes were then traversed and the distance from the last PVJ was used to determine the probability of a new PVJ. A PVJ was stochastically added to a branch segment if a randomly generated number was less than the PVJ probability at that position. A short transmurally oriented segment was then added midway along the branch at the end of which a PVJ was formed.

An efficient ray-tracing method to transmurally extend the Purkinje network from the PVJs to a certain percentage of the wall depth was implemented within *meshtool*. For every given PVJ in the Purkinje network, a single-paired intersection point on the next geometric surface is found along a trajectory defined by the endocardial surface normal at that given PVJ. The distance between paired points is then scaled by $s_{\mathrm {\rho }} \in [0,1]$ to attain an intermediate point within the ventricular wall and a branch is constructed. To generate heterogeneity, lengths of all transmurally penetrating branches are then randomized using the same Gaussian distribution spread as determined by $\sigma$.

The His-bundle and fascicular network within Ref. 25 was replaced with an adaptable representation using ${\mathcal {X}}$. Namely, $\mathbf {b}_{\mathrm {lv,sf}}$, $\mathbf {b}_{\mathrm {lv,af}}$, $\mathbf {b}_{\mathrm {lv,pf}}$ were connected to the LV branch initiation site $\mathbf {b}_{\mathrm {h,lv}}$. The fascicles pertaining to EASs of $\mathbf {b}_{\mathrm {rv,sf}}$ and $\mathbf {b}_{\mathrm {rv,mod}}$ were first connected to midway point $\mathbf {b}_{\mathrm {h,rv,mid}}$ before reaching the RV branch initiation site $\mathbf {b}_{\mathrm {h,rv}}$. This gives more flexibility in the RV bundle branch as it is known to reach the RV apex.^34^ Left and right bundle branches were joined to a bifurcation site $\mathbf {b}_{\mathrm{h,1}}$ that was ultimately connected to the exit site of the atrio-ventricular node $\mathbf {b}_{\mathrm{h,0}}$. All bifurcation points were defined based on UVCs. In total, all entities relating to growth of the Purkinje-based representation can then be combined into the parameter vector $\varvec{\omega }_{\mathrm {purkinje}}$ given as

$$\begin{aligned} \varvec{\omega }_{\mathrm {purkinje}} = \left( \delta _{\mathrm {cov}},\;\; \mu ,\, \sigma ,\, r,\, \psi ,\;\; \mathbf {b}_{\mathrm{h,0}},\, \mathbf {b}_{\mathrm{h,1}},\, \mathbf {b}_{\mathrm {h,lv}},\, \mathbf {b}_{\mathrm {h,rv}},\, \mathbf {b}_{\mathrm {h,rv,mid}} \right) . \end{aligned}$$

### Parameter Assignment

Actual parameter values for the initial HPS configurations and general EP during sinus rhythm were selected to generate the highest agreement with reported human physiology within the literature and to generate similarity to the clinically-measured 12 lead ECG of the subject,^13^ as well as ensure correspondence between model representations. Prescribed parameters for the two HPS models within $\varvec{\omega }_{\mathrm {purkinje}}$ and $\varvec{\omega }_{\mathrm {fascicle}}$ are summarized in Table 2. Sizing of the root locations in the fascicular-based model were intended to modulate only the earliest onset of breakthrough into the general myocardium from the HPS. Sizing, growth, and branching parameters of the Purkinje system were assigned to generate a realistic branching architecture that spanned a large portion of the SE layer to generate the highest agreement with reported physiology.^27^ All bifurcation points within the Purkinje model were placed basally and sub-endocardially along the septal midline with the exception of $\mathbf {b}_{\mathrm {h,rv,mid}}$ that was placed apically in the RV septum to ensure consorted activation.^27^,^34^ To modulate the same activation pattern within the fascicular-based model, activation timings defined in $\mathbf {t}_{\mathrm {his}}$ were prescribed values corresponding to timings of the EASs in the Purkinje model computed after simulation during sinus rhythm as described later in ‘Response under Sinus Rhythm and Right Ventricular Apical Pacing’. Timings are therefore relative to the start of activation from the initial node $\mathbf {b}_{h,0}$. An illustrative overview of both HPS model representations and associated parameters is shown in Fig. 1.

**Table 2:** Parameter settings assigned to generate agreement between Purkinje and fascicular-based HPS model representations.

Fascicular Representaion ($\varvec{\omega }_{\mathrm {fascicle}}$)		
Entity	Parameter	Value
Timings of earliest activation sites	$t_{\mathrm{lv,af}}$	${19.86}{\hbox { ms}}$
	$t_{\mathrm{lv,pf}}$	${26.25}{\hbox { ms}}$
	$t_{\mathrm{lv,sf}}$	${24.81}{\hbox { ms}}$
	$t_{\mathrm{rv,sf}}$	${37.30}{\hbox { ms}}$
	$t_{\mathrm{rv,mod}}$	${41.94}{\hbox { ms}}$
Sizing	$\delta _{{\mathrm{rad}}}$	0.05

Purkinje Representation ($\varvec{\omega }_{\mathrm {purkinje}}$)					
Entity	Parameter	Value			
		UVC Coordinates			
		*z*	$\rho$	$\varphi$	$\nu$
His-bundle bifurcation	$\mathbf {b}_{\mathrm{h,0}}$	1.00	0.50	0.00	−1
	$\mathbf {b}_{\mathrm{h,1}}$	0.90	0.50	0.00	−1
	$\mathbf {b}_{\mathrm {h,rv}}$	0.85	0.2	0.21	−1
	$\mathbf {b}_{\mathrm {h,lv}}$	0.85	0.8	0.21	−1
	$\mathbf {b}_{\mathrm {h,rv,mid}}$	0.3	0.8	-0.2	−1
Sizing	$\delta _{{\mathrm{rad}}}$	0.6			
Growth	*r*	0.25			
	$\psi$	${20.00}{^{\circ }}$			
Branching	$\mu$	${2100.00}\,{\mu \hbox {m}}$			
	$\sigma$	0.4			

### Response under Sinus Rhythm and Right Ventricular Apical Pacing

The response of both HPS representations were explored under healthy sinus rhythm and under RV apical pacing to investigate differences. A healthy sinus rhythm was first simulated for the Purkinje and fascicular-based representations using respective prescribed values defined in Table 2 for $\varvec{\omega }_{\mathrm {purkinje}}$ or $\varvec{\omega }_{\mathrm {fascicle}}$. In both representations, root locations as specified by $\varvec{\omega }_{\mathrm {EAS}}$ were utilized. During RV apical pacing, activation was initiated at a site located apically on the RV endocardium pertaining to a UVC coordinate of $\mathbf {b}_{ \mathrm {pace}} = (0.3, 0, 0, +1) \in D_{_{\mathrm {UVC}}}$. Within the fascicular implementation, the SE layer was kept intact to replicate retrograde activation within the Purkinje network.

Simulations were conducted using an efficient and clinically-compatible forward ECG model of cardiac EP as detailed in full in Ref. 13. The model combines the Mitchell-Schaeffer ionic model^18^ to represent cellular dynamics, the reaction-eikonal model (R-E)^19^ to model action potential propagation, and a lead-field based approach^23^ to generate potentials at each electrode placement. All simulations were conducted for a duration of ${150}{\hbox { ms}}$. Simulated 12 lead ECGs were low-pass filtered at 60 Hz, aligned, and scaled in terms of amplitude uniformly across all leads to a single heart beat of the measured 12 lead ECG as performed in Ref. 13. As no measured data was available during pacing for the single subject, ECGs were scaled in amplitude by the same value as prescribed during sinus rhythm.

Parameters relating to ventricular EP were based on physiological assumptions and previous work. Principal conduction velocity of ${0.70}\,{\hbox {m s}^{-1}}$ was applied within the general myocardium.^29^ In the Purkinje-based representation, the myocardial conduction velocity of ${0.70}\,{\hbox {m s}^{-1}}$ was also utilized within the SE layer as it was assumed that the activation stems purely from the dense Purkinje network with a higher prescribed conduction velocity of ${3.70}\,{\hbox {m s}^{-1}}$ along the Purkinje fibers.^16^,^29^ To modulate the Purkinje network in the fascicule-based represenation, the same higher conduction velocity of ${3.70}\,{\hbox {m s}^{-1}}$ was prescribed within the SE layer. Conductivities within the volume torso conductor were assigned nominal values according to Ref. 17 and conductive anisotropy within the ventricles according to Ref. 24.

The Purkinje network was assigned an anterograde and retrograde delay of ${8}{\hbox { ms}}$ and ${3}{\hbox { ms}}$, respectively.^12^ The entire network was discretized with a spatial resolution of ${500}\,{\mu \hbox {m}}$ resulting in approximately 133k node and 145k branches.

## Results

### Automated Generation of the His–Purkinje System

**Figure 2:**
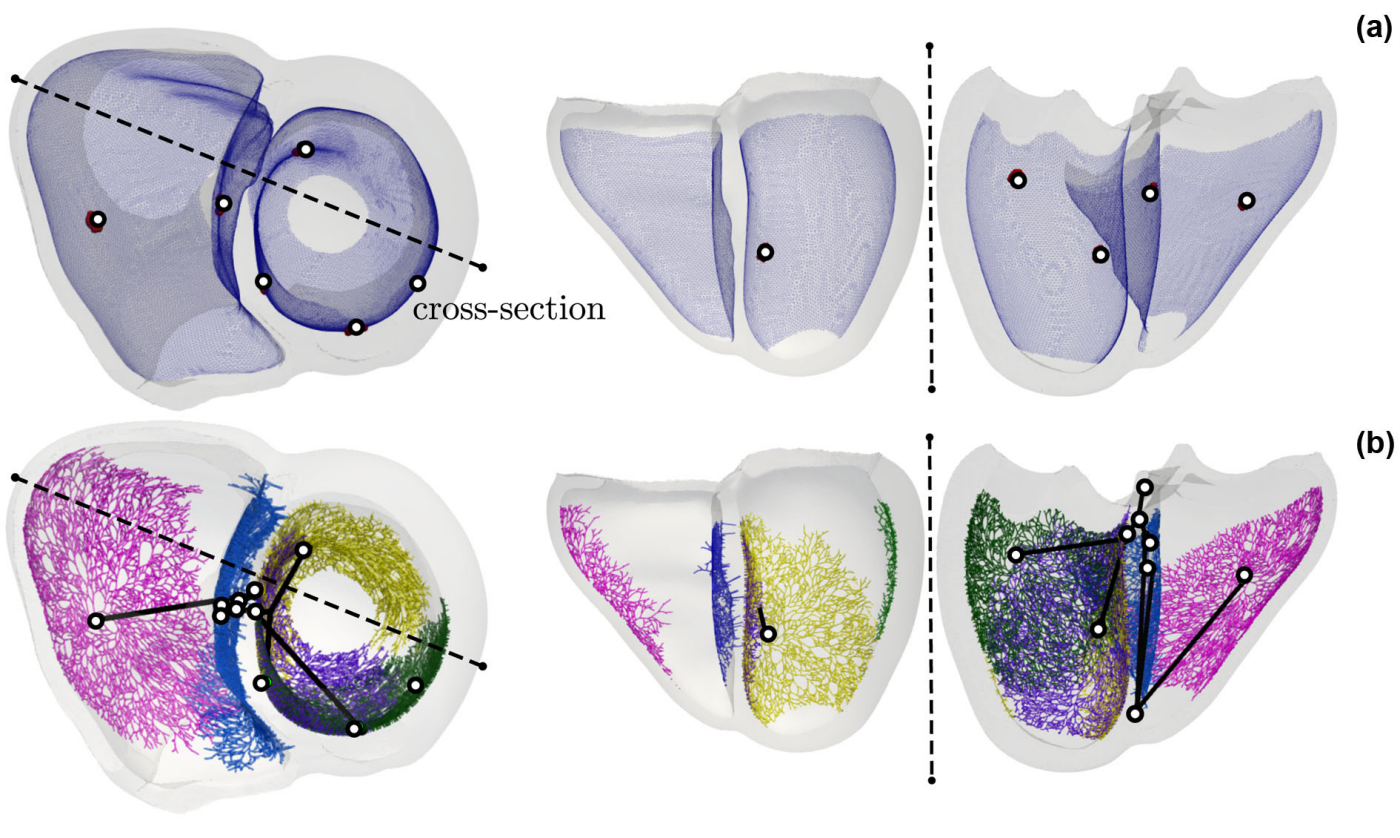
Comparison between the fasicular (a) and Purkinje-based (b) models of the HPS system initialized using $\varvec{\omega }_{\mathrm {purkinje}}$ and $\varvec{\omega }_{\mathrm {fascicle}}$, respectively. (a) Fascicular representation using discs comprises a fast-conducting SE layer with root locations as depicted corresponding to the EASs. (b) Purkinje fascicles pertaining to the RV moderator band (magenta), RV septum (blue), LV anterior (green), LV septal (purple), and LV posterior (yellow) were grown from root locations for each EAS to span the entirety of the SE layer. A His-bundle system connecting these root locations is constructed (black).

An abstract definition of a fascicle-based HPS encoded by $\varvec{\omega }_{\mathrm {fascicle}}$ (Fig. 2), previously shown to generate an activation sequence compatible with the measured ECG,^13^ was used to define a matching Purkinje-based HPS encoded in $\varvec{\omega }_{\mathrm {purkinje}}$. Respective resulting parameters are given in Table 2, and the generated anatomical HPS architectures as visualized in Fig. 2.

Ventricular activation and concomitant ECGs were simulated for both HPS representations under healthy sinus rhythm and RV apical pacing. A summary of timings relevant to construction and simulation for both $\varvec{\omega }_{\mathrm {purkinje}}$ and $\varvec{\omega }_{\mathrm {fascicle}}$ are provided in Table 3. Construction time based on $\varvec{\omega }_{\mathrm {fascicle}}$ was orders of magnitude shorter compared to using $\varvec{\omega }_{\mathrm {purkinje}}$, with 11 s and 680 s, respectively. Differences in simulation costs were less significant, by a factor of $\sim 6$, and were largely attributable to extra solver costs with $\varvec{\omega }_{\mathrm {purkinje}}$ (see  Table 3).

**Table 3:** Timings in seconds corresponding to the construction and simulation of both model representations. Simulations were executed using 16 compute cores.

	Fascicular	Purkinje
Setup of His–Purkinje system	11.0	680.3
Lead field computation	90.2	109.3
ECG forward model		
Simulation setup	8.9	29.3
Solver time	2.0	26.6
Total	10.9	55.9

### Sinus Rhythm

**Figure 3:**
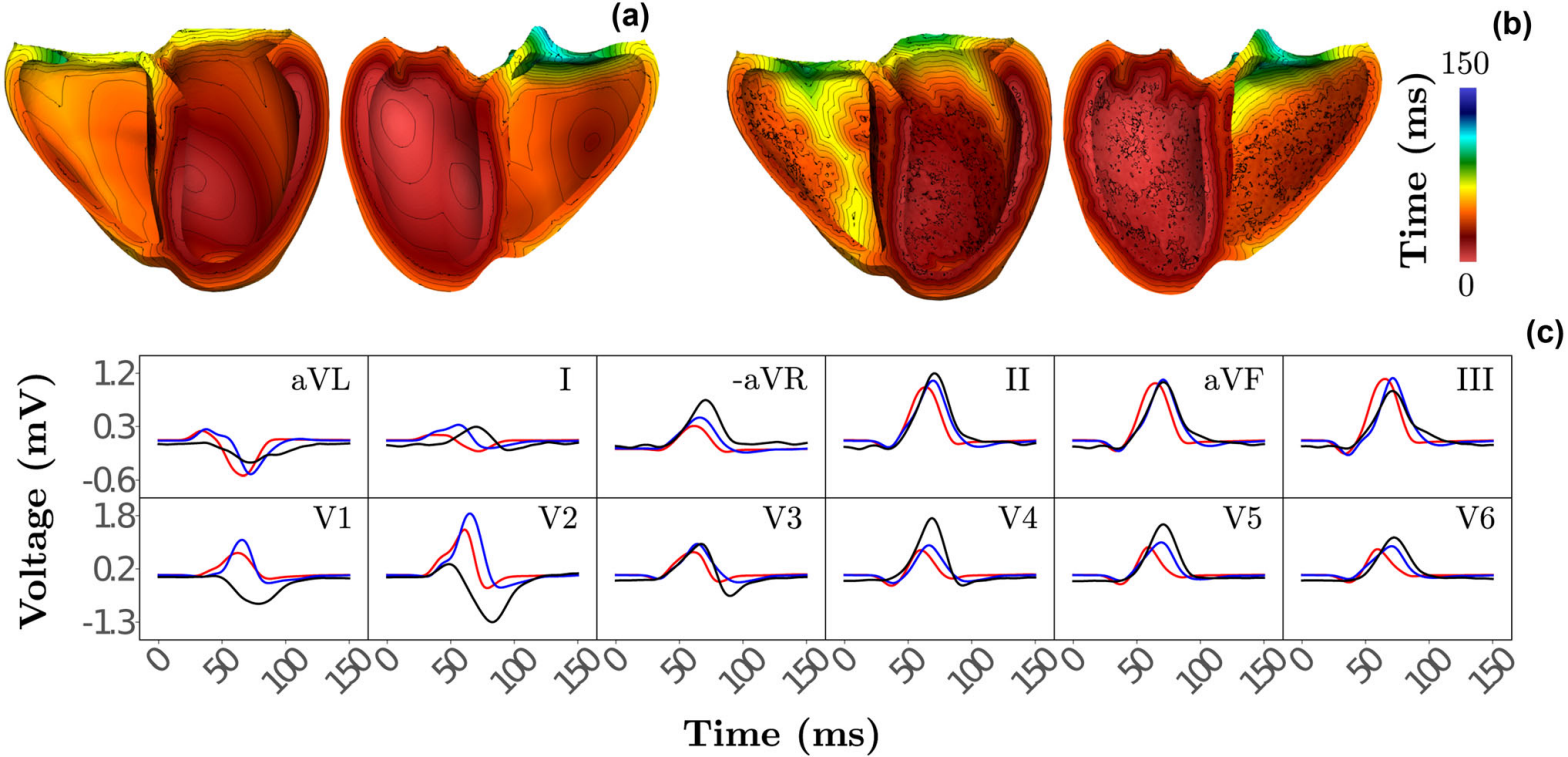
Comparison of both HPS representations during healthy sinus rhythm with orthodromic propagation in the HPS and anterograde activation of the ventricles. Ventricular activation maps of (a) fascicular-based and (b) Purkinje-based model are shown with isocontour lines every ${5}{\hbox { ms}}$ time interval. (c) Simulated 12 lead ECG resulting from the fascicular (red) and Purkinje (blue) model alongside the measured ECG (black) of the given subject.

Activation patterns under sinus rhythm with orthodromic propagation in the HPS and anterograde activation of the ventricles are shown in (Figs. 3a and 3b). Overall, the induced patterns appeared qualitatively very similar, albeit the $\varvec{\omega }_{\mathrm {purkinje}}$ pattern appeared patchier, owing to the discrete nature of the Purkinje network architecture, in contrast to the smooth nature of the SE layer (Figs. 3a and 3b). Delayed activation within the posterior RV free wall and towards the LV base was also observed. Regardless of differences in activation pattern, comparable ECG morphology was attained between the HPS representations. As previously shown^13^, agreement to the measured ECG (Fig. 3) was quite good, with greatest disagreement occurring in leads V1 and V2.

### Right Ventricular Apical Pacing

**Figure 4:**
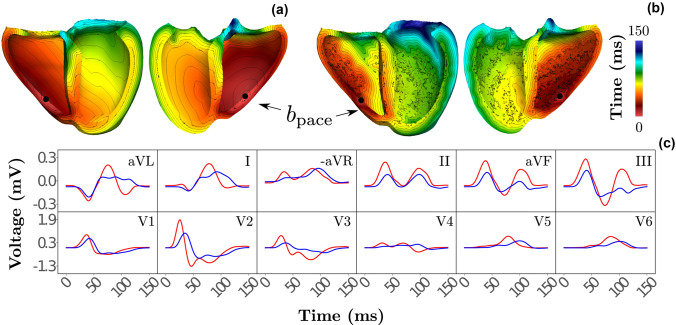
Response of both HPS models to RV apical pacing. (a, b) Ventricular activation maps obtained with (a) fascicular-based and (b) Purkinje-based models are shown with isocontour lines indicating 5 ms time intervals. Pacing location is also indicated. (c) Simulated 12 lead ECG resulting from the fascicle-based (red) and Purkinje-based (blue) models.

Due to fundamental differences between the $\varvec{\omega }_{\mathrm {purkinje}}$ and $\varvec{\omega }_{\mathrm {fascicle}}$ representation of the HPS more striking discrepancies between these are anticipated in scenarios where the HPS is activated retrogradely, with antidromic propagation within the HPS, as it may be the case under RV apical pacing. Indeed, more prominent differences were witnessed, as activation of the bulk LV in the Purkinje-based setup was delayed markedly. Opposite activation patterns were also observed within the septum, where a larger area of the LV septum activated noticeably earlier in the fascicle-based setup. Morphological ECG differences in peak amplitudes and general morphology were most apparent in the later segments of leads aVL, I, III, and V3 (Fig. 4c).

## Discussion

In this study we introduce an automated two stage approach for generating a personalized HPS for use in digital twinning pipelines. First, a simple fascicle-based HPS model representing only key emergent features such as EASs and fast SE conduction as surrogates of fascicular junctions and the Purkinje network was parameterized, aiming to minimize the misfit with the measured 12 lead ECG of the single subject.^13^ Subsequently, the fascicular-based HPS is automatically replaced by a topologically realistic Purkinje-based model of the HPS. Compared to simpler fascicle-based HPS models, a Purkinje-based model offers distinct advantages. These Purkinje-based models better represent the underlying physiology, and, thus, are able to replicate activation sequences under all scenarios beyond sinus rhythm. Further, the artificial timed stimulation at EASs and associated stimulation-induced alterations in ECG morphology are avoided. Both representations are defined by parameter vectors, $\varvec{\omega }_{\mathrm {fascicle}}$ and $\varvec{\omega }_{\mathrm {purkinje}}$, where parameters are functions of space that can be manipulated to detail ventricular EP. Feasibility of the approach is demonstrated and equivalence between the HPS model representations is investigated by comparing activation patterns under sinus rhythm and RV apical pacing. These sequences interrogate the full spectrum of interactions between HPS and ventricles by inducing orthodromic and antidromic propagation in the HPS, as well as anterograde and retrograde activation of ventricles and HPS, respectively. The topologically realistic HPS preserves the ECG fit quite well under sinus conditions. Overall, qualitatively this was also the case for RV pacing, albeit more striking differences in ECG morphology and magnitudes were witnessed.

### Automated Generation of the His–Purkinje System

Previous studies have attempted patient-specific parameterization of the HPS,^4^,^5^,^21^ primarily using endocardial mapping data. Independent of the procedures used for fitting ventricular activation to observed ECGs or endocardial mapping data, a larger number of simulations have to be performed within an optimization loop, requiring the iterative unattended adjustment of the HPS . Fascicle-based models offer clear advantages there, owing to their simplicity, easy manipulation, and the lower construction and simulation costs (Table 3). However, once emergent features are identified, a more physiological representation of the HPS is preferred that is able to represent ventricular EP under a broader range of scenarios beyond sinus rhythm. Therefore, there is a clear need for replacing the fascicle-based models after fitting, without affecting the ventricular activation sequence and concomitant ECG in a significant way. Here, we describe an automated workflow for replacing a fascicular-based model of the HPS by a topologically realistic Purkinje-based HPS, by growing HPS networks and interconnecting these to fascicles, branches and a His bundle.

A key prerequisite to preserving ECG features is the functional equivalence between the HPS models such that both models yield essentially the same ventricular activation sequence under sinus conditions. This requires topological similarity. Both HPS models share the exact same locations of the EASs and the instants of activation of these are matched, either by tuning the length of branches to scale the distance between the bundle bifurcation after the His segment, or, simpler, by adjusting conduction velocity in branches and fascicles. The fast-conducting SE layer in the fascicle-based model can be considered a surrogate of the Purkinje network and, thus, serves to bound the growth of the network within the SE. In our implementation , slight discrepancies in the extent of fast-conducting SE coverage existed the two representations (Fig. 2). While both models protrude transmurally from the endocardium to the same extent, the fast-conducting SE domain within the fascicular-based setup is continuous and spans the entirety of the endocardium within the specified apicobasal and transmural limits. The Purkinje network exhibits a patchy nature and has limited coverage based on the number and placement of the purkinje ventricular junctions within each network.

Our reported Purkinje model includes a number of differences and improvements relative to previous approaches^4^,^14^,^21^,^25^,^31^: (i) user-independent modulation of all model parameters defined within $\varvec{\omega }_{\mathrm {purkinje}}$, (ii) abstract definition of the His-bundle system, and (iii) transmural penetration of the network into the depth of the ventricular wall, and (iv) updates in the methods of Ref. 25 to generate more realistic branching. While this increased flexibility, computational costs of construction and simulations increased. Sizing and transmural extent of the Purkinje-networks, dictated by $\delta _{\mathrm {cov}}$ and $s_{{ \rho }}$, inherently play the largest role in increased computational cost as these parameters relate directly to the number of nodes and branches in the resultant Purkinje network. Assuming that the HPS is constrained to the smooth SE surface ($\delta _{{ \rho }}=0$), like all previously reported implementations, the computational cost could be significantly reduced.

### Parameter Considerations

Close agreement of the simulated 12 lead ECG during sinus rhythm with the measured clinical 12 lead ECG of the given subject as seen in Fig. 3, suggests that the parameter vectors $\varvec{\omega }_{\mathrm {EAS}}$, $\varvec{\omega }_{\mathrm {purkinje}}$ and $\varvec{\omega }_{\mathrm {fascicle}}$ can encapsulate the predominant factors relating to ventricular EP. Furthermore, timings of the EASs (Table 3), as applied indirectly through the placement of the bifurcation points in the Purkinje-based implementation and directly through $\mathbf {t}_{\mathrm {his}}$ in the fascicular-based model, agree with reported values.^11^ However, discrepancies may still exist from the underlying subject EP as primarily observed within the precordial leads V1 and V2 in Fig. 3.

Sensitivity analysis and model verification, while outside the scope of the current study, is therefore needed to better understand the influence of assumptions about the HPS configuration and general EP parameter settings on resultant ECG morphology. Impacts on the number, location and timings of the EASs summarized within $\varvec{\omega }_{\mathrm {EAS}}$ may be significant,^8^ as well as other selected parameters relating to the conduction velocities and conductive anistropy within the ventricles,^26^ and conductivities within the torso volume-conductor model.^17^ Inter-subject variability in these particular parameters might also be high and should be assessed in additional patient anatomies. Furthermore, deviation from true patient anatomy introduced during anatomical model segmentation and registration,^22^,^30^ or deviation from the actual placement of electrodes for recording 12 lead ECGs^15^ may also influence ECG morphology.

### Sinus Rhythm

Overall qualitative agreement in ventricular activation and the 12 lead ECG between HPS under sinus rhythm was satisfactory. Some quantitative discrepancies were nevertheless observed. As the EASs in both model representations activated at the same time instances, the architecture of the His-Bundle system stemming from the placement of bifurcation points, as well as the placement of EASs, did not contribute to observed differences. Thus, discrepancies primarily stemmed from substitution of the branching Purkinje networks with a fast-conducting SE layer. First, the surrogate representation of the Purkinje network in the fascicle-based HPS, modelled as a fast-conducting SE layer, was not fully covered by the grown Purkinje networks (see Fig. 2).

This was manifested by prolonged activation within regions lacking a Purkinje network (Fig. 2b) coinciding with an increase in QRS duration (Fig. 3c). Further, the SE layer was modeled as an anisotropic continuum that activated the SE layer faster along the fibers than in the transverse direction, as evidenced by the more ellipsoidal shape of endocardial activation isochrones (see Fig. 3a). In contrast, the discrete Purkinje network led to a rather isotropic spread of activation and, thus, activation isochrones of rather circular shape (see Fig. 3b). In combination with assumed anterograde activation delays of ${8}{\hbox { ms}}$^3^ this led to a more complex source distribution within the SE layer. These differences can be better visualized within the Supplementary Video S1.

### Right Ventricular Apical Pacing

As anticipated, apical pacing in the RV led to more pronounced differences between the HPS models. Retrograde activation of the Purkinje network by the ventricular myocardium close to the moderator band fascicle led to antidromic propagation in the Purkinje network of the RV which quickly activated the RV septal fascicle. The Purkinje network in the LV was activated, initially, via trans-septal wavefronts that retrogradely activated the apical Purkinje network in the LV and, later, through the left bundle branch, leading to orthodromic propagation in the portion of the network that is activated by the posterior and septal fascicles. The fascicle-based HPS led to a comparable activation of the RV, but marked differences in septal and LV activation were witnessed (see Fig. 4c and Supplementary Video S2). Overall, the fast-conducting SE layer of the fascicle-based HPS led to a much faster activation of the LV myocardium than the Purkinje-based model. Despite these differences in ventricular activation, the resulting ECGs qualitatively share morphological features. As such, a fascicle-based HPS model may also serve as plausible surrogate of a Purkinje network in scenarios beyond sinus rhythm where the Purkinje network is activated both in anterograde and retrograde direction, where propagation inside the HPS occurs in both orthodromic and antidromic directions. These differences are illustrated in the Supplementary Video S2.

## Supplementary Information

Below is the link to the electronic supplementary material.

Supplementary file1 (PDF 130 kb)

Supplementary file2 (MP4 1887 kb)

Supplementary file3 (MP4 1550 kb)
