# Supplementary material for: Automated Framework for the Inclusion of a His–Purkinje System in Cardiac Digital Twins of Ventricular Electrophysiology
Source: Ann Biomed Eng. 2021 Aug 24;49(12):3143–53. doi: 10.1007/s10439-021-02825-9 (PMC8671274; doi:10.1007/s10439-021-02825-9)
Supplement: Supplementary file 1 — Supplementary file1 (PDF 130 kb) [file 10439_2021_2825_MOESM1_ESM.pdf]

Supplementary material for: Automated Framework for the Inclusion of a His-Purkinje System in Cardiac Digital Twins of Ventricular Electrophysiology

Karli Gillette[a,b,*], Matthias A.F. Gsell[a], Julien Bouyssier[c], Anton J. Prassl[a], Aurel Neic[d], Edward J. Vigmond[c], Gernot Plank[a,b,*]

[a]*Institute of Biophysics, Medical University of Graz, Graz, Austria*
[b]*BioTechMed-Graz, Graz, Austria*
[c]*LIRYC Electrophysiology and Heart Modeling Institute, Bordeaux Foundation, Pessac, France*
[d]*NumeriCor Gmbh, Graz, Austria*

S1: Video of Membrane Voltages during Sinus Rhythm

Qualitative comparison of the membrane voltages for the fascicular-based model (**A.**) and the Purkinje-based model (**B.**) during ventricular activation is shown under sinus conditions. Please see attached supplemental content: *sinus_results_vm.mp*4

S2: Video of Membrane Voltages during RV Apical Pacing

Membrane voltages in the ventricular myocardium for both the fascicular-based model (**A.**) and the Purkinje-based model (**B.**) elucidating differences in activation during RV apical pacing. Please see attached supplemental content: *rv_apical_pacing_results_vm.mp*4

*Corresponding author
Email address: gernot.plank@medunigraz.at (Gernot Plank)

Abbreviations

CDT Cardiac Digital Twin

EAS Earliest Activation Site

ECG Electrocardiogram

EP Electrophysiology

HPS His-purkinje System

LV Left Ventricle

MRI Magnetic Resonance Imaging

PVJ Purkinje Ventricular Junction

RV Right Ventricle

SE Subendocardial

UVC Universal Ventricular Coordinates
